# Association between Dental and Cardiovascular Diseases: A Systematic Review

**DOI:** 10.31083/j.rcm2406159

**Published:** 2023-06-06

**Authors:** Louis Hardan, Anthony Matta, Rim Bourgi, Carlos Enrique Cuevas-Suárez, Walter Devoto, Maciej Zarow, Natalia Jakubowicz, Francisco Campelo-Parada, Meyer Elbaz, Didier Carrié, Jerome Roncalli

**Affiliations:** ^1^Department of Restorative Dentistry, School of Dentistry, Saint-Joseph University, 1107 2180 Beirut, Lebanon; ^2^Department of Cardiology, Institute CARDIOMET, University Hospital of Toulouse, 31059 Toulouse, France; ^3^Faculty of Medicine, Holy Spirit University of Kaslik, 42160 Jounieh, Lebanon; ^4^Dental Materials Laboratory, Academic Area of Dentistry, Autonomous University of Hidalgo State, 42160 San Agustín Tlaxiaca, Mexico; ^5^Independent Researcher, 16030 Sestri Levante, Italy; ^6^Private Practice, “NZOZ SPS Dentist'' Dental Clinic and Postgraduate Course Centre, 30-033 Cracow, Poland

**Keywords:** cardiovascular disease, clinical trials, dental, infection, tooth disease

## Abstract

**Background:**

The link between dental, infective and obstructive cardiovascular diseases is debatable.

**Aim:**

To systematically review the literature to assess the association between dental conditions and development of cardiovascular disease.

**Methods:**

The systematic review was conducted following the PRISMA guidelines using PubMed (Medline), Web of Science, Scopus, EMBASE and SciELO.

**Results:**

Out of 6680 records, 82 articles were eligible for inclusion after reviewing titles and abstracts. No association between dental disease and cardiovascular disease has been observed in 10 studies while a potential link has been suggested by the remaining trials. Tooth loss and periodontitis are the main evaluated oral conditions while coronary artery disease, stroke, atherosclerosis and myocardial infarction represent the major cardiovascular events. The interaction between these two clinical entities is based on direct mechanism mediated by systemic inflammatory response, leakage of cytokines and endothelial cells invasion by oral pathogens and indirect mechanism mediated by common risk factors or confounders.

**Conclusions:**

It seems that tooth loss, periodontitis and poor oral hygiene increase the risk of atherosclerotic cardiovascular events, and subsequently oral health care professionals could contribute to public health cardiovascular control efforts.

## 1. Introduction

Despite the recommendations on aggressive management of cardiovascular risk factors for primary and secondary prevention in parallel to the impressive progression of the available medical and interventional therapeutic strategies, cardiovascular diseases (CVD) are still the main cause of death in the developed and developing countries [1, 2]. CVD includes a broad spectrum of infective and obstructive diseases like endocarditis, myocarditis, pericarditis, acute coronary syndrome including myocardial infarction, chronic coronary artery disease (CAD), stroke and peripheral artery disease. In the acute setting of cardiovascular events or during the follow-up of patients suffering from chronic coronary syndrome, medical practitioners systematically screened for the classical risk factors like smoking, diabetes mellitus, dyslipidemia, systemic hypertension, family history of CAD, and obesity [3]. However, searching for the potentially cardiogenic atypical factors and understanding how they could affect the cardiovascular system may minimize the burden of CVD on the economic and health systems respectively [4]. For decades, researchers have been concerned by the link between oral disease and heart disease. Data from literature are conflicting and heterogeneous. Up to date, it is unclear whether the linking between these two diseases is a direct connection based on pathophysiological mechanisms making periodontal disease as independent predictor of CVD or indirect connection since these two entities commonly share multiple risk factors like smoking and unhealthy diet [5, 6]. People with oral disease are at higher risk for stroke, heart attack, and serious cardiovascular events [7, 8, 9, 10]. Periodontal disease, vertical bone lesions, endodontic disease, dental caries, dental infection were considered as dental conditions with some risk of entrapping a relationship with CVD [11, 12, 13, 14, 15]. Study findings revealed a positive association between the reduction in teeth number, abdominal aortic calcification [16], ischemic events [9, 10] and cardiovascular mortality [7, 8]. In addition, lesion originating from endodontic disease is able to trigger a systemic illness [17, 18]. Simultaneously, patients with CVD significantly present a lower teeth number and poor oral hygiene [19].

Considering that oral health status could directly influence the incidence, pathophysiology, and course of CVD, it is important to summarize the literature to better describe this potential association and the mechanisms which can explain this link. Herein, this paper aimed to systematically review the association between dental conditions and development of CVD.

## 2. Materials and Methods

### 2.1 Study Design

A systematic review of clinical trials that examine the association of dental disease and CVD disease was performed according to the guidelines of the Preferred Reporting Items for Systematic Reviews and Meta-Analyses (PRISMA statement) [20]. The registration protocol was carried out in Open Science Framework with the registration number 0000-0002-2759-8984. The following PICOS strategy was used: population, human; intervention, dental disease, control, patients without CVD; outcome: CVD; type of study, observational studies, clinical trials. The research question was: Does the presence of dental disease is associated with the development of CVD?

### 2.2 Search Strategy

An unlimited literature search was performed by two independent reviewers (RB and CECS) until November 11th, 2021 using PubMed (MEDLINE), Web of Science, Scopus, EMBASE and SciELO. The MeSH search terms in the previously cited databases are summarized in Table 1. All research studies were imported into Rayyan QCRI platform.

**Table 1.** **Keywords used in search strategy**.

Search strategy	
# 1	Caries OR Dental health OR Periodontal disease OR Periapical Disease OR Tooth Diseases OR Oral pathology OR dental infection OR oral infection OR Dental Pulp Disease OR Oral Health
# 2	Heart Disease OR Vascular Disease OR Coronary Artery Disease OR Coronary heart disease OR Atherothrombotic cardiovascular disease
# 3	Clinical trials OR Controlled Clinical Trial OR Retrospective Studies OR Randomized Controlled Trial OR Prospective clinical trial OR Retrospective Study OR Prospective Studies OR Prospective Study OR Clinical Trial OR Randomized clinical trial
# 4	# 1 AND # 2 AND # 3

### 2.3 Inclusion Criteria

The title and abstract of each recognized manuscript were examined by two independent reviewers (RB and CECS) to determine if the article should be considered for full-text review according to the following eligibility criteria: (1) Case-control and cross-sectional studies, cohorts, and randomized clinical trials reporting the relationship of any cardiovascular condition with the presence of any oral disease; (2) studies where the presence of an oral disease was clinically diagnosed; (3) studies where CVD was clearly defined; and (4) peer-reviewed articles published in the English, Spanish or Portuguese languages.

### 2.4 Exclusion Criteria

Case reports, case series, pilot studies, expert opinions, conference abstracts and reviews were excluded. In case of disagreements at the time of the collection of the papers for the full-text review, they were resolved by discussion and agreement by a third reviewer (LH).

### 2.5 Data Extraction

Data of interest were extracted from the enrolled manuscripts via the Microsoft Office Excel 2019 program (Microsoft Corporation, Redmond, WA, USA) and subsequently placed on a standardized form. Two reviewers (RB and LH), who received training in this software, performed data analysis. The extracted data from each manuscript include author names, year of publication, study type, number of participants, oral health condition, CVD, biomarkers, and principal outcomes.

## 3. Results

**Fig. 1.**
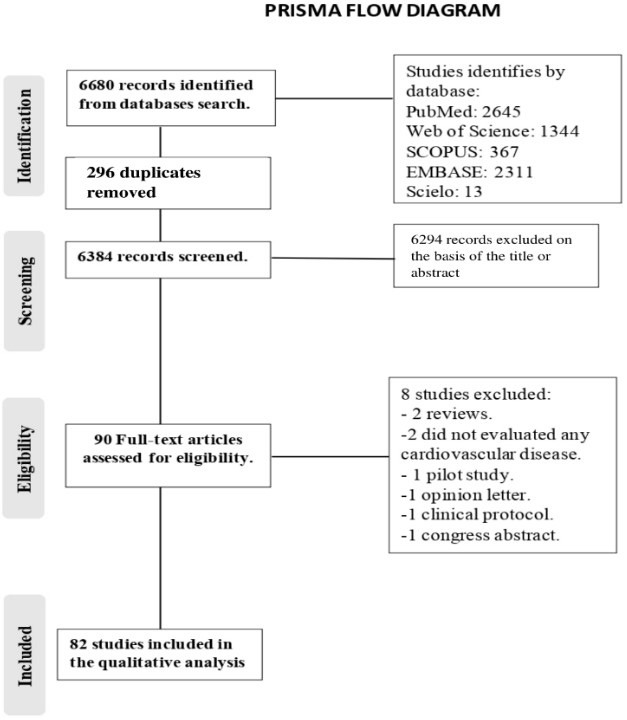
**Search flowchart according to the PRISMA Statement**.

The search resulted in the retrieval of 6680 records. After removal of duplicates, 6384 articles were screened, and 6294 were excluded based on the title or abstract. A total of 90 full-text articles were assessed for eligibility [20, 21, 22, 23, 24, 25, 26, 27, 28, 29, 30, 31, 32, 33, 34, 35, 36, 37, 38, 39, 40, 41, 42, 43, 44, 45, 46, 47, 48, 49, 50, 51, 52, 53, 54, 55, 56, 57, 58, 59, 60, 61, 62, 63, 64, 65, 66, 67, 68, 69, 70, 71, 72, 73, 74, 75, 76, 77, 78, 79, 80, 81, 82, 83, 84, 85, 86, 87, 88, 89, 90, 91, 92, 93, 94, 95, 96, 97, 98, 99, 100, 101, 102, 103, 104, 105, 106, 107, 108, 109, 110]. Of these, eight were not considered for the qualitative analysis: two were reviews [24, 54], two did not evaluated any CVD [51, 63], one was a pilot intervention study [32], one was an editorial letter [37], one was a clinical protocol [56] and one was a congress abstract [67]. Finally, 82 manuscripts were considered for the qualitative analysis (Fig. 1). Characteristics of the included studies in this systematic review are summarized in Table 2 (Ref. [21, 22, 23, 25, 26, 27, 28, 29, 30, 31, 33, 34, 35, 36, 38, 39, 40, 41, 42, 43, 44, 45, 46, 47, 48, 49, 50, 52, 53, 55, 57, 58, 59, 60, 61, 62, 64, 65, 66, 68, 69, 70, 71, 72, 73, 74, 75, 76, 77, 78, 79, 80, 81, 82, 83, 84, 85, 86, 87, 88, 89, 90, 91, 92, 93, 94, 95, 96, 97, 98, 99, 100, 101, 102, 103, 104, 105, 106, 107, 108, 109, 110]).

**Table 2.** **Characteristics of the included studies**.

Study (year)	Type of study	Number of participants	Oral health condition	Cardiovascular disease	Biomarkers measured	Principal outcomes
Abnet 2005 [21]	Cohort	29,584	Tooth loss	Heart disease	Not measured	Individuals with greater median number of teeth lost had statistically significant increased risk of death from heart disease and stroke of 28% and 12%, respectively.
				Stroke		
Aoyama 2018 [22]	Clinical trial	897	Periodontal conditions	Coronary heart disease	Not measured	The coronary artery disease patients generally had worse oral condition than the non-coronary artery disease patients.
Batty 2018 [23]	Cohort	975,685	Tooth loss	Coronary heart disease	Not measured	There was a moderate, positive association between tooth loss and coronary artery disease.
Berent 2011 [25]	Cross-sectional study	466	Periodontal conditions	Coronary heart disease	Not measured	Periodontal disease is a potentially risk factor for developing coronary artery disease.
Boillot 2015 [26]	Prospective cohort	841	Periodontal infections	Cardiovascular disease	Quantitative assessment of 11 bacterial species. Secretory phospholipase A2 (s-PLA2) and Lipoprotein-associated PLA2	The relationship between periodontal microbiota and vascular diseases maybe linked to Greater s-PLA2 activity at higher tertiles.
Boillot 2016 [27]	Prospective multicentre observational study	975	Periodontal conditions	Acute myocardial infarction	Levels of IgG and IgA against *Porphyromonas gingivalis, Aggregatibacter actinomycetemcomitans, Prevotella intermedia and Tannerella forsythia*	In the setting of prior myocardial infarction, the risk of major cardiovascular events was not increased in association with the levels of circulating periodontopathogens antibodies.
Bokhari 2014 [28]	Cross-sectional study	317	Periodontitis	Coronary heart disease	Bleeding on probing, probing depth, clinical attachment level, high-sensitivity C-reactive protein, fibrinogen and white blood cells	Bleeding on probing is strongly associated with systemic high-sensitivity C-reactive protein.
Caplan 2009 [29]	Cohort	6651	Endodontic therapy	Coronary heart disease	Not measured	Greater self-reported history of endodontic therapy was more likely to have coronary artery disease than those reporting no history of endodontic therapy.
Cho 2020 [30]	Cohort	514,832	Periodontal disease	Peripheral arterial disease	Not measured	The hazard ratio of peripheral arterial disease in the periodontitis group compared with that in the control group was 1.15.
Cho 2021 [31]	Retrospective cohort study	298,128	Periodontal Disease	Acute Myocardial Infarction and Stroke	Not measured	Severe periodontal disease increased total acute myocardial infarction events by 4.3%, total stroke events by 1.4%, and the total nonfatal major adverse cardiovascular events by 1.6%.
D’Aiuto 2006 [33]	Parallel-arm randomized clinical trial	40	Periodontal infections	Cardiovascular disease	C-reactive protein	Periodontal treatment reduces systemic inflammatory markers and improves lipid profiles.
					Interleukin-6	
					Leukocyte counts	
					High-density	
					lipoprotein cholesterol	
Desvarieux 2003 [34]	Prospective population-based cohort	711	Periodontal Disease and Tooth Loss	Carotid Artery Plaque	Serum total cholesterol	Tooth loss is related to subclinical atherosclerosis.
					High-density	
					lipoprotein cholesterol	
					Low-density	
					lipoprotein cholesterol	
Glodny 2013 [35]	Cross-sectional	292	Dental caries and chronic apical periodontitis	Atherosclerosis	Not measured	Dental caries, pulpal caries, and chronic apical periodontitis are associated positively with aortic atherosclerotic burden.
Gurkan 2014 [36]	Case Control	32	Periodontitis	Coronary Artery Ectasia	Not measured	There is an association between periodontitis and coronary artery ectasia.
Howell 2001 [38]	Double-blind placebo-controlled trial	22,037	Periodontal Disease	Nonfatal myocardial infarction	Not measured	Periodontal disease is not an independent predictor of subsequent cardiovascular disease.
				Nonfatal stroke		
				Cardiovascular death		
Hujoel 2000 [39]	Prospective cohort	8032	Periodontal disease	Coronary heart disease	Total serum cholesterol level	There is no evidence of a causal association between periodontal disease and coronary artery disease.
Hung 2003 [40]	Prospective cohort	45,136	Tooth loss	Peripheral arterial disease	High-density	Tooth loss is associated with peripheral arterial disease, especially among men with periodontal diseases.
					lipoprotein cholesterol	
Janket 2013 [41]	Case Control	256	Tooth loss	Coronary artery disease	Low-density	The number and quality of remaining teeth impact on cardiovascular survival.
					lipoprotein cholesterol	
					C-Reactive Protein	
Johansson 2014 [42]	Case Control	161	Periodontal disease	Coronary artery disease	Non measured	There is not a significant association coronary artery disease and periodontal status.
Joshipura 1996 [43]	Prospective cohort	44,119	Tooth loss and periodontal disease	Coronary heart disease	Non measured	Periodontal disease was not associated with coronary heart disease. Tooth loss may increase the risk of the later one.
Khouja 2019 [44]	Prospective cohort	320	Periodontal disease	Coronary artery disease	Non measured	Periodontal disease was a significant predictor of coronary heart disease among current smokers with diabetes.
Kotronia 2020 [45]	Cross-sectional	5222	Periodontal disease	Cardiovascular disease	C-reactive	Tooth loss was associated with some inflammatory, haemostatic and cardiac biomarkers.
			Tooth loss		protein	
			Dry mouth		interleukin-6	
					tissue plasminogen activator von Willebrand Factor	
					fibrin D-dimer	
					high sensitivity Troponin T	
					N-terminal pro-brain natriuretic peptide	
Li 2010 [46]	Prospective cohort study	10,958	Periodontal disease	Cardiovascular disease	Total cholesterol	Tooth loss was related to an increased risk of death due to cardiovascular disease.
			Tooth loss		High-density	
					lipoprotein cholesterol	
Lockhart 2009 [47]	Double-blind randomized placebo controlled study	194	Periodontal disease	Infective endocarditis–related bacteremia	Non measured	Bacteremia is associated with poor oral hygiene and gingival bleeding after toothbrushing.
Montenegro 2019 [48]	Single-blind parallel-design randomized controlled trial	82	Severe chronic periodontitis	Coronary artery disease	C-reactive	Periodontal therapy lower levels of systemic inflammation.
					Glucose	
					Glycated hemoglobin	
					Triglycerides	
					Total cholesterol	
					High-density lipoprotein cholesterol	
					low-density lipoprotein cholesterol	
					IL-1β	
					IL-6	
					IL-8	
					IL-10	
					IFN-γ	
					TNF-α	
Morrison 1999 [49]	Retrospective cohort study	12,795	Periodontal disease	Coronary heart	Non measured	Poor dental health is associated with an increased risk of fatal coronary heart disease.
				Cerebrovascular diseases		
Nakib 2004 [50]	Cohort	6931	Periodontitis	Coronary artery calcification	Total cholesterol	Periodontitis is not associated with coronary artery calcification.
					Triglycerides	
					High-density	
					lipoprotein cholesterol	
Napora 2016 [52]	Cross-sectional study	119	Periodontal disease	Risk cardiovascular parameters	Non measured	The most significant periodontal parameter in relation to the progression of atherosclerosis and left ventricle hypertrophy was shown to be clinical attachment loss.
Offenbacher 2009 [53]	Multicentered Randomized Controlled Trial	303	Periodontal status	Systemic levels of high-sensitivity C-reactive protein	High-sensitivity C-reactive protein	Periodontal therapy may lower hs-CRP levels among non-obese cardiovascular patients.
Peng 2017 [55]	Retrospective Cohort Study	15,195	Periodontal disease	Myocardial infarction	Non measured	Periodontal therapy lowers the rate of myocardial infarction and heart failure.
				Heart failure Stroke		
Saffi 2018 [57]	Randomized parallel-design examiner-blinded controlled trial	69	Periodontal disease	Endothelial function in coronary artery disease	C-reactive protein	Periodontal treatment maintained blood concentrations of markers of vascular inflammation.
					Glucose	
					Glycated hemoglobin	
					Triglycerides	
					Total cholesterol	
					High-density lipoprotein cholesterol	
					Low-density lipoprotein cholesterol	
Seinost 2020 [58]	three-armed observer-blinded randomized controlled trial	90	Severe periodontitis	Peripheral arterial disease	C-reactive protein	Periodontal treatment did not reduce vascular inflammation in patients with peripheral arterial disease.
					IL-6	
					Leukocyte	
					HbA1c	
					Total cholesterol	
					HDL cholesterol	
					LDL cholesterol	
					Triglycerides	
Shearer 2018 [59]	Cohort	1139	Periodontitis	Markers of cardiometabolic risk	glycated haemoglobin	Periodontitis was not associated with markers of cardiometabolic risk.
					triglycerides	
					HDL cholesterol	
Sia 2021 [60]	Cohort	13,402	Periodontitis	Incidental valvular heart disease	Non measured	Periodontitis was associated with a significant risk for valvular heart disease. Treatment of periodontal disease reduced the risk.
Spahr 2006 [61]	Case control study	1315	Periodontal infection	Coronary heart disease	Non measured	An association between periodontitis and presence of coronary heart disease was found.
Subha 2017 [62]	Double blinded randomized clinical trial	45	Generalized severe periodontitis	Serum markers of cardiovascular diseases	C-Reactive Protein	Periodontal treatment reduced the level of serum markers of cardiovascular diseases.
					Total Cholesterol	
					High Density Lipid	
					Low Density Lipid	
					Triglycerides	
Tiensripojamarn 2021 [64]	Cohort	1850	Periodontitis	Coronary heart disease	Non measured	Severe periodontitis is associated with an increased incidence of coronary heart disease.
				Stroke		
Tonetti 2009 [65]	Exclude. Review article.					
Ueno 2012 [66]	Case-control	573	Periodontitis	Coronary heart disease	Antibody levels of periodontopathic bacteria	Elevated antibody levels to periodontopathic bacteria is associated with an increased risk of coronary artery disease.
Vedin 2014 [68]	Cohort	15,828	Periodontitis	Coronary heart disease	Low-density lipoprotein cholesterol	indicators of periodontal disease were common in the population with coronary heart disease.
					Fasting p-glucose	
					High sensitivity C-reactive protein	
Vedin 2015 [69]	Cohort	15,456	Tooth loss	Coronary heart disease	Non measured	Tooth loss predicted adverse cardiovascular outcomes.
Vernon 2011 [70]	Prospective longitudinal study	43	Periodontal disease	Cardiovascular disease	Carotid artery intima media thickness	Periodontal disease may contribute to cardiovascular risk.
					Brachial artery flow-mediated dilation	
Wilson 2018 [71]	Prospective cohort	20,133	Number of teeth, severity of dental plaque and the presence of oral lesions.	Incident myocardial infarction	Non measured	Poor oral health is associated with a slightly increased risk of myocardial infarction.
Xu 2011 [72]	Cohort	10,849	Periodontal disease	Heart disease	High sensitivity C-reactive protein	An association between periodontal disease and cardiovascular disease and all-cause mortality was found.
				Cerebrovascular diseases	White cell count	
					Fibrinogen	
Gomes 2015 [73]	Longitudinal Study	278 dentate participants	Apical periodontitis (AP)	Cardiovascular events (CVE) including angina, myocardial infarction, and cardiovascular-related death	The total number of AP and RCT sites was determined from panoramic radiographs. EB was calculated as the sum of AP and RCT sites. Oral inflammatory burden (OIB) was calculated combining periodontal disease and EB	EB in midlife was an autonomous predictor of CVE.
			Root canal treatment (RCT)			
			Endodontic burden (EB)			
Isola 2019 [74]	Clinical trials	143 patients	Periodontitis	Ischemic heart disease	Levels of vitamin C, antioxidants, and C-reactive protein (hs-CRP) were assessed with a commercially available kit	Patients with ischemic heart disease and periodontitis plus ischemic heart disease presented lower levels of salivary and serum vitamin C compared to healthy subjects and periodontitis patients. hs-CRP was a significant predictor of decreased salivary and serum vitamin C levels.
			Gingival health			
Pasqualini 2012 [75]	A case-controlled clinical trial	100 participants	Oral infections	Acute myocardial infarction or unstable angina	Non-measured	Chronic oral diseases might increase the risk of coronary artery disease (CHD) and could be a risk factor for CHD.
Lee 2019 [76]	Observational study	Eighty-eight patients	Periodontal disease	Coronary artery disease	White blood cell count.	Tooth loss was linked with the presence of obstructive CAD in patients experiencing coronary assessment.
					Haemoglobin	
					Total cholesterol	
					LDL cholesterol	
					HDL cholesterol	
					Triglyceride	
					Fasting glucose	
					Glycated haemoglobin	
Emingil 2000 [77]	Clinical study	120 patients	Periodontal disease	Acute myocardial infarction and chronic coronary heart disease	Missing teeth, restorations, probing depth (PD) and bleeding on probing (BOP) were recorded. Blood samples were taken on admission for measurements of serum total cholesterol, triglycerides, high density lipoprotein cholesterol (HDL-cholesterol), low density lipoprotein cholesterol (LDL-cholesterol), and fasting blood glucose level	Periodontal disease might be linked with acute myocardial infarction.
Stenman 2009 [78]	A cross-sectional study	n = 1056	Chronic periodontitis (CP)	Ischemic heart disease (IHD)	Number of missing teeth, age, body mass index, waist/hip ratio, life satisfaction, hypertension, and levels of cholesterol and triglycerides	Periodontitis did not seem to have a statistically significant relationship with IHD. The number of missing teeth showed a strong association with IHD.
Eno Belinga 2018 [79]	A prospective observational study	558 patients	Periodontal disease	Cardiovascular diseases	Non measured	A link between periodontal diseases and cardiovascular diseases were highlighted in this study.
Petersen 2013 [80]	Retroscpective	531 patients	Chronic apical periodontitis (CAP)	Atherosclerosis	The volume of the aortic atherosclerotic burden	A correlation between CAP without endodontic treatment and aortic atherosclerotic burden was found.
			Endodontic therapy			
Cotti 2011 [81]	Prospective	40 mens	AP	Atherosclerosis	All subjects underwent dental examination and complete cardiac assessment: physical examination, electrocardiogram, conventional and tissue Doppler echocardiography, and measurement of endothelial flow reserve (EFR). The following laboratory parameters were tested: interleukins -1, -2, and -6 (IL-1, IL-2, IL-6), tumor necrosis factor alpha, and asymmetrical dimethylarginine (ADMA)	Increased ADMA levels and their relationship with poor EFR and increased IL-2 might suggest the existence of an early endothelial dysfunction in young adults with AP.
Byon 2020 [82]	Retrospective Matched	52,425 participants	Periodontitis	Atherosclerotic	Propensity score matching	The presence of periodontitis increased the risk of atherosclerosis.
	Cohort Study					
Çalapkorur 2016 [83]	Cross-sectional study	60 patients	Periodontal disease	Peripheral arterial disease (PAD)	Ankle–brachial index values	Periodontitis did increase the odds ratio for having PAD.
Chen 2012 [84]	Prospective cohort design	1 million persons	Tooth scaling	Cardiovascular events	Propensity score matching	Tooth scaling was associated with a reduced risk for upcoming cardiovascular events.
Vedin 2017 [85]	Clinical trial	15,828 participants	Tooth loss	Coronary heart disease	Tooth loss levels	An association between tooth loss and coronary heart disease was found.
					Linear and Cox regression models	
Gugnani 2021 [86]	Parallel-group, unicentric, randomized, assessor- blinded, superiority trial	48 patients	Severe periodontitis	Endothelial function	Cytokine levels	Treatment of severe periodontitis improved the endothelial function.
Santos‐Paul 2019 [87]	Single‐center, observational study	409 patients	Periodontitis	Cardiovascular mortality	Demographic, clinical, and laboratory characteristics	Treatment of periodontitis reduced the incidence of cardiovascular events.
Oliver 2018 [88]	Retrospective	428 children	Poor oral health	Infective endocarditis	Medical and dental records	Age, socio-economic status, and enamel defects were associated with caries experience, not severity of cardiac diagnoses.
Mariott 2013 [89]	Controlled clinical trial	64 patients	CP	Heart disease	C-reactive protein (CRP) and interleukin-6 (IL-6) levels	Treatment of periodontal disease reduced the risk of heart disease.
Persson 2003 [90]	Clinical research	80 subjects	CP	Acute myocardial infarction (AMI)	Non-measured	Patients who at routine dental visits establish sign of bone loss nearby numerous teeth can probably be recognized as being at risk for future AMI.
Byun 2020 [91]	Cross-Sectional Analysis	173,209 participants	Periodontitis	Cerebral stroke/ischemic heart disease	History of hypertension, diabetes mellitus, hyperlipidemia cerebral stroke (hemorrhagic or ischemic), ischemic heart disease (angina or myocardial infarction), and periodontitis. Their body mass index, smoking habit, alcohol intake nutritional intake, and income were recorded	An association between periodontitis and cardiovascular disease existed.
DeStefano 1993 [92]	Prospective cohort study	9760 subjects	Dental disease	Coronary heart disease	Incidence of mortality or admission to hospital because of coronary heart disease; total mortality	Dental disease was associated with an increased risk of coronary heart disease.
Naudi 2006 [93]	Retrospective study	195 subjects	Dental health and preventive practices of child patients	Congenital heart disease	Oral examination	Children with cardiac problems should be identified in early infancy through liaison with medical colleagues and the family offered all the preventive advice and regular dental care necessary to prevent dental disease.
Findler 2013 [94]	A retrospective observational comparison study	54 patients	Dental disease	Refractory heart failure	Non measured	This study provided essential dental treatment for severe heart failure patients with special attention to their medical problems and the use of medications and supporting means to prevent health-compromising situations is recommended.
Skilton 2011 [95]	A randomized, controlled trial study	450 adults	Periodontal disease	Vascular health and inflammation	Inflammatory mediators (IL-1, IL-6, TNF)	Periodontal disease was related to CVD.
Saffi 2013 [96]	A randomized, parallel design, examiner blinded, controlled clinical trial	100 participants	Periodontal therapy	Coronary artery disease	C-reactive protein, endothelial function, lipids and proinflammatory biomarkers	A relation between periodontal treatment and coronary artery disease existed.
Caúla 2014 [97]	Randomized clinical trial	64 patients	Severe chronic periodontitis	Cardiovascular risk markers	Inflammatory markers	A reduction in the level of inflammatory biomarkers and improvement in lipid profiles were observed after medical treatment in patients with chronic periodontitis.
Gunupati, 2011 [98]	Cross-sectional randomized clinical study	72 patients	CP	Acute myocardial infarction	Serum IgG and IgM aCL antibodies	The phase I periodontal therapy altered levels of serum IgG and IgM aCL antibodies in patients with AMI associated with chronic periodontitis.
Ide 2013 [99]	Randomized clinical study	39 patients	CP	Acute-phase inflammatory and vascular responses	Circulating levels of cardiovascular and systemic inflammatory markers	Improvement in periodontal health did not influence the levels of vascular markers.
Tüter 2007 [100]	Clinical trial study	36 patients	CP	Coronary artery disease	Gingival crevicular fluid (GCF) levels of matrix metalloproteinase (MMP) -1, -8, -13 and on serum levels of high-sensitivity C-reactive protein (hscrp) and lipid fractions	A combination produced statistically significant benefits in both local periodontal disease (GI and PD) and systemic biomarkers.
Cowan 2020 [101]	Multicenter population-based prospec- tive cohort study	6638 participants	Endodontic infection (EI)	Risk of coronary heart disease (CHD), ischemic stroke (IS), heart failure (HF), or venous thromboembolism (VTE)	Cox- proportional hazards regression models were used to estimate hazard ratios	Our results do not support an independent association between ET and development of CHD, IS, HF, or VTE.
Koppolu 2013 [102]	Clinical study	40 subjects	Periodontitis	CVD	CRP & TNF-α	Periodontal disease treatment significantly reduced the level of inflammatory biomarkers reflecting a possible relationship periodontitis and the pathogenesis of CVD.
Toregeani 2015 [103]	Clinical research	98 patients	Periodontal disease	Atherosclerosis	Carotid intima-media thickness (CIMT) and expression of laboratory markers	Preventing periodontal disease helped in preventing atherosclerosis.
Hoke 2011 [104]	Prospective	411 patients	Dental disease	Atherosclerosis	Hyperlipidaemia	Dental status and oral hygiene were associated with mortality in patients with carotid atherosclerosis regardless of conventional cardiovascular risk factors.
					Total cholesterol (mg/dL)	
					Low-density lipoprotein cholesterol High-density lipoprotein cholesterol Glycated haemoglobin	
Huang 2018 [105]	Cohort	3613 patients	Periodontal disease	Atherosclerotic vascular disease	Cox proportional hazard model	A reduction in cardiovascular risk was observed after an intensive treatment of periodontal disease.
Bresolin 2013 [106]	Prospective clinical study	33 children	Periodontal treatment	Atherosclerosis	Lipid profiles and inflammatory markers	Periodontal treatments were effective in children with congenital heart disease.
Chou 2015 [107]	A population-based follow-Up Study	32,504 adult patients	Treated Periodontitis	Cardiovascular Events	Gender, hyperlipidemia, hypertension, and diabetes mellitus	Periodontitis was linked to an increased risk of cardiovascular events.
Bokhari 2012 [108]	Randomized controlled trial	317 patients	Non-surgical periodontal therapy	Coronary heart disease	Serum CRP levels	In the context of coexisting periodontitis and coronary artery disease, non-surgical mechanical periodontal approach decreased the levels of hs C-reactive protein, fibrinogen and white blood cells.
					Fibrinogen and white blood cells	
Holmlund 2010 [109]	Cohort	7674 Subjects	Oral health	Cardiovascular mortality	Evaluation of the relation between remaining teeth, grading of periodontal disease, deep of periodontal pockets, bleeding on probe and cause of mortality	CVD is linked to oral health status.
Janket 2014 [110]	Cohort	256 consecutive coronary artery disease patients	Oral infection	Cardiovascular mortality	C-reactive protein, fibrinogen	An improvement of 27% in CVD survival has been observed after incrementation of 10 teeth.

Regarding oral health conditions, most of the studies evaluated the tooth loss and periodontal disease while the assessed cardiovascular events were stroke, CAD, acute myocardial infarction, peripheral artery disease, and atherosclerotic disease. Findings from the included studies in this systematic review were in favor of an association between the poor oral conditions (especially for the reduction in tooth number and periodontal disease) and the incidence of cardiovascular events (particularly for stroke and CAD). Also, the presence of periodontal disease was associated with higher level of inflammatory biomarkers.

Out of the 84 studies, ten studies failed to reveal a link between gum disease and CVD [5, 27, 38, 39, 42, 50, 58, 59, 99, 101]. Two studies declined the connection between periodontitis and ischemic heart disease while it showed an association between tooth loss and ischemic heart disease [43, 78]. Otherwise, tooth loss was associated with increased in cardiac mortality [21, 46], death from stroke [21], CAD [23, 85, 76], adverse cardiovascular outcomes [69], atherosclerosis [34], peripheral artery disease [40], inflammatory, hemostatic, and cardiac biomarkers [45] and impaired cardiovascular survival [41]. Periodontal disease was associated with CAD [25, 44, 64, 96], peripheral artery disease [30, 83], acute myocardial infarction [31, 77], stroke [31], adverse cardiovascular events [31, 70, 72, 107], coronary artery ectasia [36], valvular heart disease [60], cardiovascular disease [79, 91, 95, 102] and atherosclerosis [82]. In parallel, patients with CAD express higher level of periodontal disease indicators and have poor oral conditions [22, 68]. A reduction in systemic inflammatory and cardiac biomarkers [33, 48, 57, 62], improvement in lipid profile [33], decreasing in prevalence of acute myocardial infarction [55], heart failure [55], cardiovascular events [87], heart disease [89] and improvement in endothelial function [86] have been observed after periodontitis treatment and good oral health care. Lastly, increment of 10 teeth from the edentulous state resulted in 27% improvement in CVD survival [110].

## 4. Discussion

**Fig. 2.**
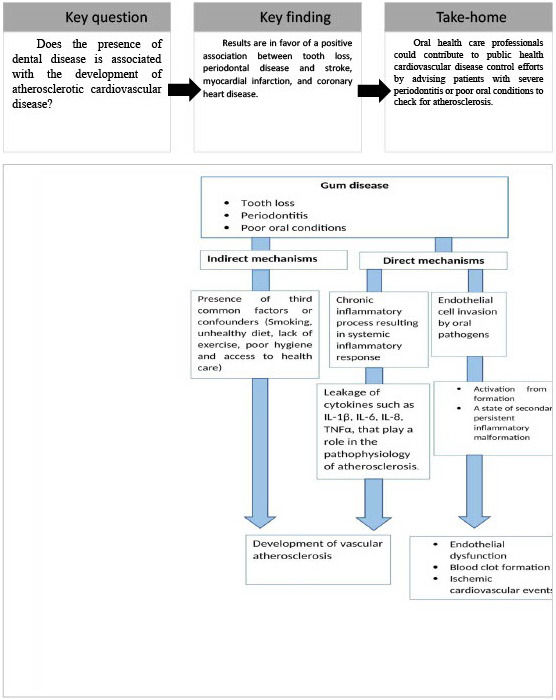
**Review’s rational and findings with the potential mechanisms linking gum disease and development of cardiovascular disease**.

In this review, the association between dental conditions and development of CVD was studied and results were in favor for a positive association between tooth loss, periodontal disease, and CVD (Fig. 2). Conventional risk factors for atherosclerosis and CAD such as smoking, diabetes, hypertension, high low-density lipoprotein (LDL) serum level, obesity, male gender, and genetic predisposition have been evidently recognized and systematically searched after cardiovascular event [111]. Smoking and familial history play the pivotal role in the development of CVD in young patients [112]. Unconventional risk factors like chronic inflammatory reactions have also been identified as predictors of CVD. When the inflammation occurs, circulating markers and hemostatic factors were diligently linked with the development of myocardial infarction [113]. Particularly, chronic oral infection was associated with chronic heart diseases [114, 115]. Dental infections were associated with an increased prevalence of heart disease [75, 116], as the oral cavity was the main site of inflammation and chronic infection, especially in cases of tooth loss and chronic periodontal diseases [75, 117]. Many studies have discovered that the long-standing inflammatory stimuli of dental infection was implicated in the pathogenesis of CVD [117, 118], though further analyses have failed to notice a strong relationship between CVD and dental infection [39, 40, 41, 42, 43, 44, 45, 46, 47, 48, 49, 50, 51, 52, 53, 54, 55, 56, 57, 58, 59, 60, 61, 62, 63, 64, 65, 66, 67, 68, 69, 70, 71, 72, 73, 74, 75, 76, 77, 78, 79, 80, 81, 82, 83, 84, 85, 86, 87, 88, 89, 90, 91, 92, 93, 94, 95, 96, 97, 98, 99, 100, 101, 102, 103, 104, 105, 106, 107, 108, 109, 110, 111, 112, 113, 114]. Oikarinen *et al*. [119] have documented a higher rate of periodontal infection among patients with CAD and Söder B *et al*. [118] have revealed that a high dental calculus score was correlated with increased incidence of angina pectoris. With regards to tooth loss and CAD and stroke risk, there are several unsolved issues [120]. The results obtained from this review suggest that there is a relationship between the number of teeth loss and the presence of cardiovascular events, like CAD, peripheral arterial disease, and increased risk of stroke death. This could be explained by the fact that first the number of tooth loss might be linked to some inflammatory, hemostatic, and cardiac biomarkers [45]. Second, one should consider that the main cause of tooth loss is dental caries, and carbohydrate intake is the chief dental caries cause. If specialist consider that carbohydrate intake is associated with increased risk of CVD and stroke, then the number of tooth loss could have indirect impact on these two last mentioned diseases [121]. Third, as the progress of tooth damage destroys periodontal tissues, thus oral microbial will accumulate into oral tissue, therefore promoting its growth and resulting in an increased risk of CVD and stroke [122]. All these factors were trustworthy for elucidating the relation between the CVD and tooth loss. Also, the release and persistence of early inflammatory biomarkers of periodontitis like TGF-β, transglutaminases and NLRP3 accelerate atherosclerotic plaque development [123]. Inflammatory proteins have been associated in several observational studies with endothelial dysfunction, atheroma plaque development and cardiovascular events [124]. Details of the biological processes that lead to the systemic inflammatory reaction in the setting of periodontal diseases are not well understood. It maybe seems that these elevations of systemic inflammatory markers occur in response to the exposure to oral bacteria. Another hypothesis considers that cytokines secreted by the inflamed periodontal tissues would cross the blood stream causing a systemic inflammation [125].

In this context studying the link between oral and cardiovascular diseases, there is another topic of great importance that is worth mentioning. It is the concern of patients taking anticoagulant for cardiovascular disease and who undergo dental procedures. The management of such as patients seems much more complicated by dentists especially in the absence of a clear implemented protocol [126].

Several hypotheses have been raised to explain the association between dental and CVD. For example, pathogens causing gingivitis and periodontitis can travel into blood vessels elsewhere in the organism resulting in inflammation of the vascular wall, parietal damage and blood clots formation [122]. This rational was supported by polymerase chain reaction detection of oral bacterial remnants like *streptococcus mutans*, *porphyromonas gingivalis*, *prevotella intermedia *and tannerella forsythia in the fatty deposits within the atherosclerotic vessels [127, 128, 129]. It remains unclear by which mechanism periodontal pathogens could influence atherosclerosis after direct endothelial cells invasion. Triggering foam cell formation or provoking a state of secondary inflammation through their intracellular persistence leading to endothelial dysfunction have been suggested [130]. Another theory depends on the body’s immune response to chronic inflammatory process that sets off a cascade of vascular damage throughout the body including heart and brain. Periodontitis activates a systematic inflammatory response that produces high levels of different cytokines like Il-1β, Il-6, Il-8 and TNF-α also playing a role in the pathophysiology of atherosclerotic vascular disease [131]. Lastly, it could be possible that gum disease and CVD are not directly connected but they may occur together in the presence of third common factor or potential confounders such as smoking, unhealthy diet, poor hygiene, lack of exercise and poor access to healthcare. Indeed, individuals without health insurance or who don’t take care of their global health are more likely to have worse oral conditions and CVD. Noteworthy that periodontal disease and CVD share genetic predisposition via at least one susceptibility locus [132, 133].

Overall, gum disease and CVD are multifactorial disorders requiring interaction between several factors and any potential contribution of one disease to the pathology of other should be carefully interpreted as many confounding variables affect both conditions. Health care professionals have to be aware of this association. Thus, dental practitioners should advise patients with severe periodontitis to check with physicians for atherosclerosis and cardiologists should insist on the importance of good oral hygiene.

## 5. Conclusion

In conclusion, the results obtained from this systematic review suggests that oral condition, especially the number of remaining teeth and the presence of periodontal disease increase the risk of cardiovascular events. It is likelihood that the association is mainly related to a chronic persistent systemic inflammatory reaction. Future research must be directed, especially randomized controlled clinical trials, with the purpose of gaining a better understanding of the link between oral and cardiac diseases.

## Supplementary Material

Supplementary material associated with this article can be found, in the online version, at https://doi.org/10.31083/j.rcm2406159.
