# Supplementary figures and images for: Association between Dental and Cardiovascular Diseases: A Systematic Review
Source: Rev Cardiovasc Med. 2023 Jun 6;24(6):159. doi: 10.31083/j.rcm2406159 (PMC11264166; doi:10.31083/j.rcm2406159)

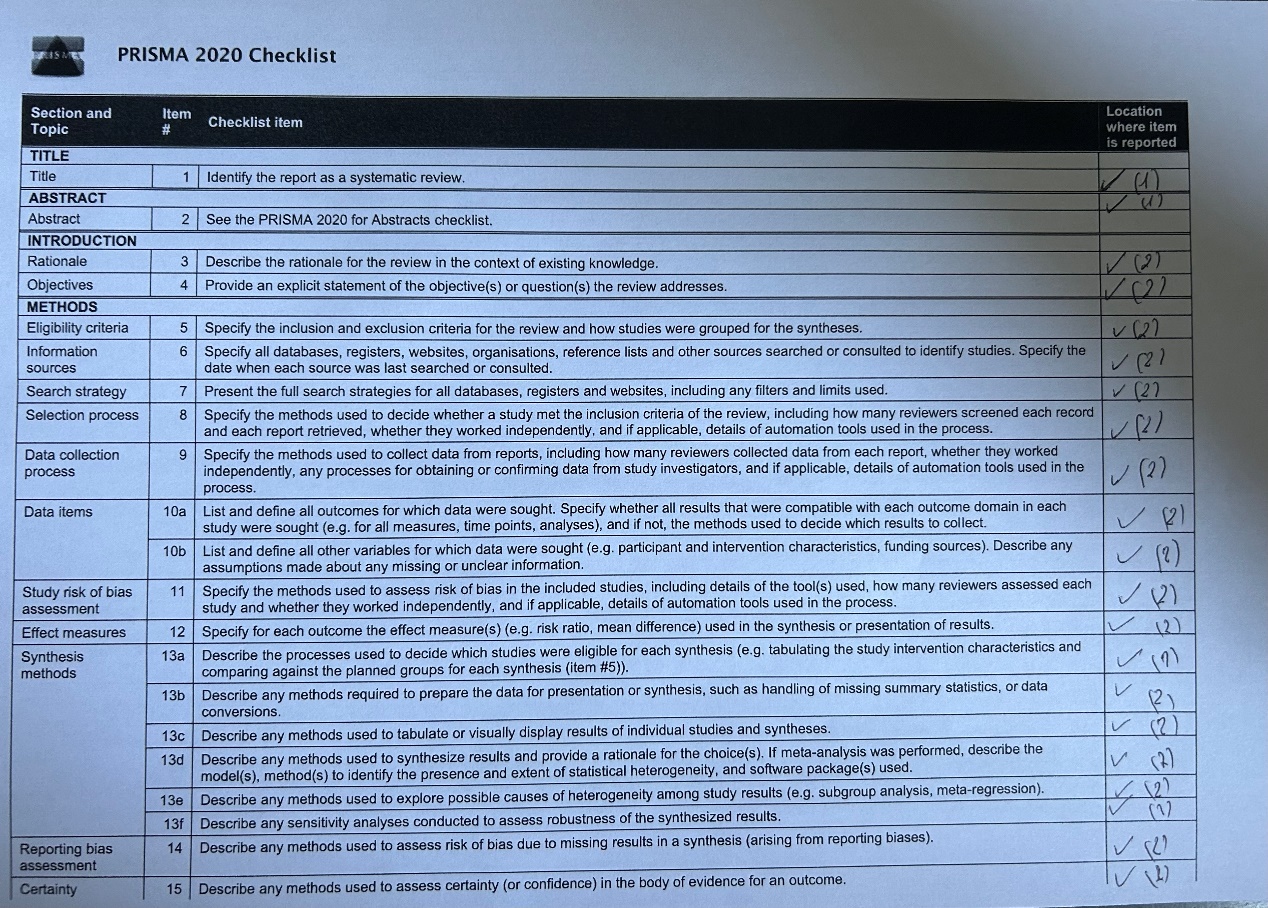

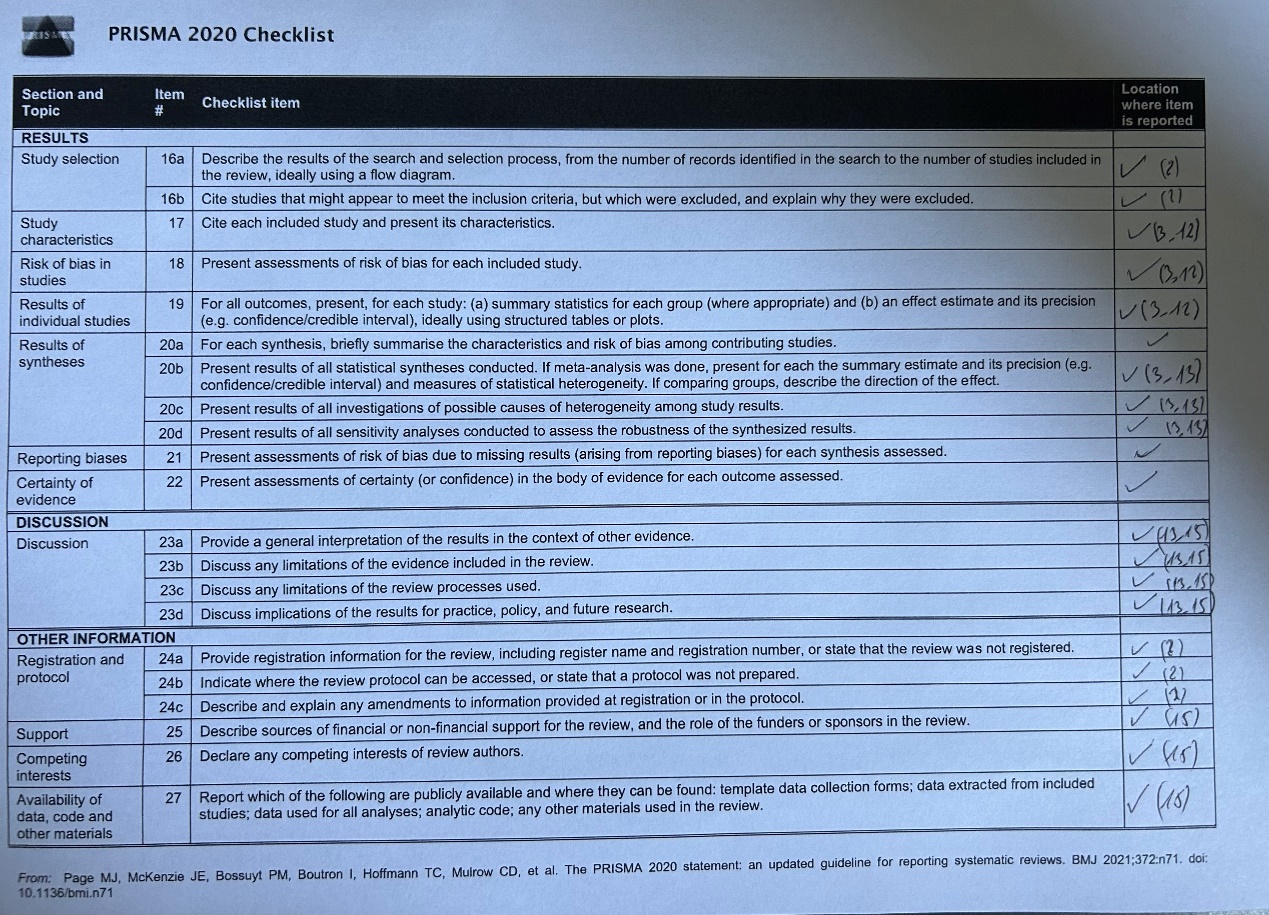

Supplement: Supplementary file 1 [file 2153-8174-24-6-159-s1.docx]
